# School Attendance Pre- and Post-Pandemic in Adolescents with Chronic Pain

**DOI:** 10.3390/children13010015

**Published:** 2025-12-20

**Authors:** Jasmine R. Berry, Leslie Sim, Cynthia Harbeck-Weber, Karen Weiss

**Affiliations:** 1 Department of Psychiatry and Psychology, Mayo Clinic, Rochester, MN 55905, USA; sim.leslie@mayo.edu (L.S.); harbeckweber.cynthia@mayo.edu (C.H.-W.); weiss.karen2@mayo.edu (K.W.); 2 Department of Pediatrics and Adolescent Medicine, Mayo Clinic, Rochester, MN 55905, USA

**Keywords:** chronic pain, adolescents, intensive interdisciplinary pain rehabilitation program, school, functional disability, internalizing symptoms

## Abstract

**Highlights:**

**What are the main findings?**

- In the post-pandemic era, youth with high-impact chronic pain are more likely to attend online or home-school than prior to the pandemic.
- Youth who attend online school are at greater risk of chronic school absenteeism and functional disability than those who attend school in person.

**What are the implications of the main findings?**

- Though the shift to online school may have helped students with high-impact chronic pain access schooling, it also increases the risk of school absence and greater disability.
- The results support evidence-based treatment for pediatric chronic pain, which emphasizes return to full functioning, including return to in-person schooling.

**Abstract:**

**Background/Objective**: The COVID-19 pandemic led to a widespread transition to online learning. Yet, it is unclear how this shift in learning format impacted school attendance in adolescents with chronic pain, a group that struggles with school absenteeism. This study compared school attendance and format (e.g., in-person, online) in patients with chronic pain attending an Intensive Interdisciplinary Pain Treatment Program (IIPT) before the pandemic to an age- and gender-matched sample of youth attending the program after the pandemic. **Methods**: Participants were 226 school aged adolescents (13–18 years; *M* age = 15.89, *SD* = 1.46) enrolled in an IIPT before and after the COVID-19 pandemic. Patients admitted before March 2020 (pre-pandemic group; *n* = 113) were compared to an age- and gender-matched group of patients who were admitted to the IIPT from November 2021 to November 2023 (post-pandemic group = 113). Upon admission to the program, participants completed validated measures of internalizing symptoms and functional disability. They also completed structured questions related to pain and school history. **Results**: Significantly more patients in the post-pandemic group were attending school through online and home-schooling options (*p* < 0.01). Youth who attended the IIPT after the pandemic missed fewer school days (*p* < 0.05) than those who attended before the pandemic. The groups did not differ in functional disability or internalizing symptoms. However, participants in the post-pandemic group who attended online school missed more school days (*p* < 0.01) and experienced greater functional disability (*p* < 0.05) than those who attended in person. **Conclusions**: The findings highlight how the COVID-19 pandemic may lead to sustained changes in the way adolescents with chronic pain attend school, with implications for increased functional disability and school dropout among this vulnerable population.

## 1. Introduction

School closures during the COVID-19 pandemic were implemented to reduce the introduction and transmission of SARS-CoV-2 [1]. Though intended as a protective measure to restrict SARS-CoV-2 infections, school closures limited students’ access to learning and peer environments, with broad implications for adolescent development [2]. Adverse effects of school closures include diminished opportunities for cognitive and social development [3], as well as reduced participation in athletics, extracurricular and other valued activities [2]. Of concern, these lost opportunities for development have been linked to problems in youth psychiatric adjustment, with distinct increases in symptoms of depression and anxiety from pre-pandemic levels [4].

It is well established that adolescents with chronic pain experience disruptions in critical domains of adolescent development including school and extracurricular activities, as well as peer and family relationships [5]. As such, school closures during the COVID-19 pandemic may have been especially harmful to these youth. In fact, adolescents with chronic pain are more likely to experience school difficulties than their pain-free counterparts, including impaired ability to cope with classroom demands, frequent absences, and decline in academic performance [6,7], with implications for psychological adjustment [8].

Given the proliferation of online learning opportunities after the COVID-19 pandemic and its potential impact on youth with chronic pain, the current study was conducted as an exploratory investigation aiming to compare a cohort of youth who attended an Intensive Interdisciplinary Pain Treatment Program (IIPT) before the COVID-19 pandemic to an age- and gender-matched cohort of youth who attended after the pandemic on school attendance and learning format (e.g., in person, online, hybrid). We also aimed to examine the relationship of school attendance and format to internalizing symptoms and functional disability in these youths. Though the study was exploratory, it was hypothesized that adolescents who attended the IIPT post-pandemic would experience greater school absenteeism as well as higher levels of anxiety, depression, and functional disability than adolescents who attended the IIPT pre-pandemic.

## 2. Materials and Methods

### 2.1. Participants

Participants included adolescent patients (*n* = 226), ages 13–18, (*M* = 15.89, *SD* = 1.46) who had been struggling with debilitating chronic pain and/or associated symptoms (e.g., dizziness, fatigue) enrolled in an IIPT. Debilitating chronic pain includes chronic pain that interferes with daily functioning, which can include school attendance or performance, social activities, extracurricular activities, physical activity, eating, sleep, driving, chores, and mood. Participants who were admitted between March 2019 and March 2020 were included in the pre-pandemic cohort (*n* = 113). These participants were consecutively admitted to the IIPT. Participants who were admitted from November 2021 to November 2023 were age- (within 1 year) and gender-matched to the pre-pandemic cohort (post-pandemic cohort; *n* = 113). There was one participant in the post-pandemic group we were not able to match: a 17-year-old male. Therefore, the final sample consisted of 113 participants in each group. Similarly to other studies on youth with chronic pain, the majority of participants identified as predominantly White/Caucasian (*n* = 203, 89%) and female (*n* = 162, 71.7%). Please see Table 1 for other demographic and patient characteristics.

### 2.2. Treatment Program

Participants were enrolled on a rolling basis throughout the year in an IIPT that consisted of 15 days of intensive outpatient services that included pain medicine, pain psychology, physical therapy, occupational therapy, and recreational therapy. Details about this program have been published elsewhere [9].

### 2.3. Procedure

Study procedures were approved by the Institutional Review Board (IRB # 004702) and patients (age 18 or older) or their parents (patient age < 18) signed informed consent to participate. Upon admission to the program, participants completed structured questions related to pain and school history including questions about school format and the number of missed days of school, as well as clinical measures. Patients responded to a structured question about the type of school they attend “What type of schooling are you enrolled in,” with response items including “regular full time, regular part time, online, homeschooled, homebound, and hybrid”. Missed days of school were self-reported by participants, “In the past 12 months, how many days of school did you miss because of your pain?”

### 2.4. Measures

Participants rated their current pain level using the Pain intensity Numerical Rating Scale (NRS), an 11-point rating scale ranging from 0 = “no pain” to 10 = “worst pain.” Youth rated symptoms of anxiety on the Spence Children’s Anxiety Scale (SCAS) [10], a well-validated 38-item self-report measure of the frequency of various symptoms associated with anxiety. Depression was measured with the Center for Epidemiological Studies Depression Scale for Children (CESD) [11], a 20-item self-report measure of depressive symptoms with acceptable reliability and validity for adolescents [12]. Finally, participants rated their perception of their functional disability using the Functional Disability Inventory (FDI) [13], a well-established, 15-item self-report measure that assesses difficulty in physical and psychosocial functioning due to health status [14].

### 2.5. Statistical Analyses

To compare differences between cohorts on pain intensity, pain duration, days of missed school, and scores on the SCAS and CESD, we conducted independent *t*-tests. We conducted a chi-square test to examine differences in type of school for participants who participated in the IIPT before the pandemic compared to after the pandemic. To explore potential differences between type of school and functioning for post-pandemic participants, we conducted a post hoc analysis using independent *t*-tests to examine differences between those who attended in person school to those who attended an online format on missed days of school, and scores on the SCAS, CESD and FDI. Post hoc analyses were not a part of the original analysis plan, and were exploratory in nature to further investigate the impact of increased availability of online schooling options post-pandemic in a youth chronic pain population.

## 3. Results

### Analyses

There were no differences between the pre- and post-pandemic groups for pain intensity (*t* (224) = −0.41, *p* = 0.34), pain duration (*t* (224) = −1.38, *p* = 0.09) or functional disability (*t* (224) = 0.44, *p* = 0.33). See Table 2. Participants in the pre-pandemic group reported significantly more missed days of school compared to the post-pandemic group (*t* (218) = 1.75, *p* <0.05). There were no significant differences between the pre- and post-pandemic groups for depressive symptoms (CESD; *t* (224) = −1.03, *p* = 0.15) or anxiety symptoms (SCAS; *t* (224) = 0.31, *p* = 0.38). See Table 2. There was a significant difference in type of school attended between pre- and post-pandemic groups (c^2^ (6, 226) = 17.64, *p* < 0.01), with an increase in online and home-schooling options post-pandemic, and a decrease in homebound and in-person, part-time schooling options post-pandemic. See Table 1 for means and percentages.

Exploratory analyses suggested that participants in the post-pandemic cohort who attended online school had significantly more missed days of school (*t* (69) = −2.78, *p* < 0.01) and higher functional disability than those attending full time in person school (FDI; *t* (69) = −1.82, *p* <0.05). There were no differences between these groups for depression symptoms (CESD; *t* (69) = −0.59, *p* = 0.28) or anxiety symptoms (SCAS; *t* (69) = −0.43, *p* = 0.33).

## 4. Discussion

The aim of this cross-sectional study was to investigate the impact of the COVID-19 pandemic on school attendance in adolescents with chronic pain attending an IIPT. As expected, adolescents attending the program after the pandemic were more likely to be enrolled in online school than those who attended the IIPT before the pandemic. Yet, compared to youth in the pre-pandemic cohort, youth in the post-pandemic cohort reported fewer missed days of school. There were no differences in internalizing symptoms or functional disability between the groups. Exploratory analyses found that youth in the post-pandemic cohort who attended online school reported more school absences and higher levels of functional disability than individuals attending who attended school in person.

Though these findings might suggest that for youth coping with chronic pain, online school facilitates school attendance without detriment to youth psychological adjustment, scores on measures of depressive symptoms were quite high in both groups. As such, ceiling effects may account for the lack of a relationship between school format and internalizing symptoms. In fact, exploratory analyses in the post-pandemic cohort show that online options were associated with a significant increase in both missed days of school and functional disability. These findings are consistent with the fear avoidance model, as online schooling options can serve as a reinforcer for school avoidance and inhibit return to functioning/increase functional disability [15].

The current study demonstrates that more youth with chronic pain in the post-pandemic era are choosing online options. However, the results also indicate this option is associated with greater school absence and higher levels of functional impairment. As such, the results support evidence-based treatment for pediatric chronic pain which emphasizes a gradual return to full functioning, including a return to in-person schooling and daily activities. Given the proliferation and widespread acceptance of online schooling options and their implications for functional disability, future research should examine ways to support youth with chronic pain in returning to in-person school.

This study is limited by the cross-sectional design, post hoc analyses, and multiple *t*-tests. Multiple comparisons increase the occurrence of finding a statistically significant result purely by chance and thus runs the risk of type I error. As this study is correlational, it is also limited in its implications, as a clear causal pathway cannot be inferred. Thus, there may be alternative explanations for the association between increased functional impairment and online schooling, such as youth with more severe impairment self-selecting into online schooling options. This study is also limited due to its ethnically and racially homogenous sample of youth with high-impact chronic pain, which limits our ability to generalize the findings to more diverse populations of youth and those with less severe symptoms and disability. Finally, the data in this study showed a clear trend toward musculoskeletal pain and conversion disorder in the post-pandemic cohort. It is not clear if this is indicative of broader secular changes or shifts in referral patterns, however this shift in primary concern could potentially impact the generalizability and outcomes of this study.

These preliminary findings raise important questions about how the COVID-19 pandemic may lead to sustained changes in the way adolescents with chronic pain attend school, potentially contributing to school drop out for youth already at risk. Future research should examine both the positive and negative effects of these format changes for adolescents who experience chronic pain/symptoms. This research will be paramount in addressing school absence and enhancing functioning in this vulnerable population.

## Figures and Tables

**Table 1:** Participant demographic and school-related characteristics.

	Pre-Pandemic		Post-Pandemic	
	*M*/*n*	*SD*/%	*M*/*n*	*SD*/%
**Age**	15.88	1.48	15.89	1.44
**Gender**				
Female	81	71.7	81	71.7
Male	28	24.8	28	24.8
Other	4	3.5	4	3.5
**Primary Condition**				
Musculoskeletal, limb, or joint pain	16	14.2	36	31.9
Orthostatic intolerance	40	35.4	5	4.4
Abdominal pain	25	22.1	22	19.5
Headache or Migraine	21	18.6	19	16.8
Conversion disorder	2	1.8	27	23.9
Complex Regional Pain Syndrome	1	0.9	4	3.5
Other	7	6.2	0	0
**Race and Ethnicity**				
White/Caucasian	101	89.4	102	90.3
Black/African American	1	0.9	1	0.9
Asian	0	0	2	1.8
American Indian/Alaska Native	1	0.9	0	0
Multi-racial	7	6.2	4	3.5
Other	2	1.8	1	0.9
Chose not to answer	1	0.9	0	0
**School Type**				
Regular full time	45	39.8	44	38.9
Regular part time	18	15.9	12	10.6
Online	21	18.6	27	23.9
Homeschool	5	4.4	9	8.0
Homebound instruction	14	12.4	3	2.7
Hybrid	9	8.0	8	7.1
Did not choose any option	1	0.9	10	8.8

**Table 2:** Differences in Pain, Missed Days of School, Functional Disability, and Internalizing Symptoms between pre- and post-pandemic cohorts.

	Pre-Pandemic		Post-Pandemic				
	*M*	*SD*	*M*	*SD*	*t* (224)	*p*	Cohen’s D
Pain Intensity	4.81	2.28	4.94	2.25	−0.41	0.34	−0.6
Pain duration	3.50	3.53	4.22	4.23	−1.38	0.09	−0.18
Missed Days of School	73.59	58.01	60.87	48.97	1.75	<0.05	0.24
FDI	26.97	10.66	26.34	10.89	0.44	0.33	0.06
CESD	28.36	14.25	30.21	12.64	−1.03	0.15	−0.14
SCAS Total Score	36.33	18.45	35.52	20.97	0.31	0.38	0.04

## Data Availability

The participants of this study did not give written consent for their data to be shared publicly, so data cannot be made available.

## Abbreviations

The following abbreviations are used in this manuscript:

IIPT	Intensive Interdisciplinary Pain Treatment
NRS	Numerical Rating Scale
CESD	Center for Epidemiological Studies Depression Scale for Children
FDI	Functional Disability Inventory
